# From policy to practice: progress towards data- and code-sharing in ecology and evolution

**DOI:** 10.1098/rspb.2025.1394

**Published:** 2025-09-17

**Authors:** Edward R. Ivimey-Cook, Alfredo Sánchez-Tójar, Ilias Berberi, Antica Culina, Dominique G. Roche, Rafaela A. Almeida, Bawan Amin, Kevin R. Bairos-Novak, Heikel Balti, Michael G. Bertram, Louis Bliard, Ilha Byrne, Ying-Chi Chan, William R. Cioffi, Quentin Corbel, Alexander D. Elsy, Katie R. N. Florko, Elliot Gould, Matthew J. Grainger, Anne E. Harshbarger, Knut Anders Hovstad, Jake M. Martin, April Robin Martinig, Giulia Masoero, Iain R. Moodie, David Moreau, Rose E. O'Dea, Matthieu Paquet, Joel L. Pick, Tuba Rizvi, Inês Silva, Birgit Szabo, Elina Takola, Eli S. J. Thoré, Wilco C. E. P. Verberk, Saras M. Windecker, Gabe Winter, Zuzana Zajková, Romy Zeiss, Nicholas Patrick Moran

**Affiliations:** ^1^ School of Biodiversity, One Health, and Veterinary Medicine, University of Glasgow, Glasgow, UK; ^2^ Department of Evolutionary Biology, Bielefeld University, Bielefeld, Germany; ^3^ Department of Biology, Carleton University, Ottawa, Ontario, Canada; ^4^ Ruder Boskovic Institute, Zagreb, Croatia; ^5^ Department of Biology and Institute of Environmental and Interdisciplinary Science, Carleton University, Ottawa, Ontario, Canada; ^6^ Laboratory of Freshwater Ecology, Evolution, and Conservation, Department of Biology, KU Leuven, Leuven, Belgium; ^7^ Faculty of Social and Behavioural Sciences, Utrecht University, Utrecht, The Netherlands; ^8^ School of the Environment, The University of Queensland, Brisbane, Queensland, Australia; ^9^ Université Marie et Louis Pasteur, CNRS, Chrono-environnement (UMR 6249), F-25000 Besançon, France; ^10^ Department of Wildlife, Fish, and Environmental Studies, Swedish University of Agricultural Sciences, Umeå, Västerbotten, Sweden; ^11^ Department of Zoology, Stockholm University, Stockholm, Sweden; ^12^ School of Biological Sciences, Monash University, Melbourne, Victoria, Australia; ^13^ Department of Evolutionary Biology and Environmental Studies, University of Zurich, Zurich, Switzerland; ^14^ Swiss Ornithological Institute, Seerose 1, 6204 Sempach, Switzerland; ^15^ Southall Environmental Associates, Aptos, California, USA; ^16^ Theoretical and Experimental Ecology Station (SETE), National Center for Scientific Research (CNRS), Moulis, France; ^17^ Department of Environmental Systems Science, ETH Zurich, Switzerland; ^18^ Freshwater Institute, Fisheries and Oceans Canada, Canada; ^19^ School of Agriculture, Food and Ecosystem Sciences, The University of Melbourne, Parkville, Victoria, Australia; ^20^ Norwegian Institute for Nature Research (NINA), P.O. Box 5685 Torgarden, Trondheim NO-7485, Norway; ^21^ Duke University Marine Laboratory, Duke University, Beaufort, North Carolina, USA; ^22^ SINTEF Ocean, Trondheim, Norway; ^23^ School of Life and Environmental Sciences, Deakin University, Geelong, Victoria, Australia; ^24^ The Okanagan Institute for Biodiversity, Resilience, and Ecosystem Services, University of British Columbia, Kelowna, British Columbia, Canada; ^25^ Evolution & Ecology Centre and School of Biological, Earth and Environmental Sciences, UNSW, Sydney, NSW, Australia; ^26^ Department of Biology, Lund University, Lund, Sweden; ^27^ School of Psychology and Centre for Brain Research, University of Auckland, Auckland, New Zealand; ^28^ Institute of Ecology and Evolution, University of Edinburgh, Edinburgh, UK; ^29^ Center for Advanced Systems Understanding (CASUS), Helmholtz-Zentrum Dresden-Rossendorf (HZDR), Görlitz, Germany; ^30^ Centre for Research on Ecology, Cognition and Behaviour of Birds, University of Ghent, Ghent, Belgium; ^31^ Department of Computational Landscape Ecology, UFZ—Helmholtz Centre for Environmental Research, Leipzig, Germany; ^32^ Laboratory of Adaptive Biodynamics, Research Unit of Environmental and Evolutionary Biology, Institute of Life, Earth, and Environment, University of Namur, Namur, Belgium; ^33^ TRANSfarm - Science, Engineering, & Technology Group, KU Leuven, Belgium; ^34^ Department of Ecology, Radboud University Nijmegen, Nijmegen, The Netherlands; ^35^ The Kids Research Institute, Perth, Australia; ^36^ School of Physics, Mathematics and Computing, University of Western Australia, Perth, Australia; ^37^ Population Ecology Group, Institute of Ecology and Evolution, Friedrich-Schiller-Universitat Jena, Jena, Germany; ^38^ Institute of Marine Sciences (ICM-CSIC), Barcelona, Spain; ^39^ Experimental Interaction Ecology, German Centre for Integrative Biodiversity Research (iDiv) Halle-Jena-Leipzig, Leipzig, Saxony, Germany; ^40^ Institute of Biology, Leipzig University, Leipzig, Germany; ^41^ Centre of Excellence for Biosecurity Risk Analysis (CEBRA), Biosciences, University of Melbourne, Parkville, Victoria, Australia

**Keywords:** open science, journal policy, reproducibility, replicability, transparency, peer review

## Abstract

Data and code are essential for ensuring the credibility of scientific results and facilitating reproducibility, areas in which journal sharing policies play a crucial role. However, in ecology and evolution, we still do not know how widespread data- and code-sharing policies are, how accessible they are, and whether journals support data and code peer review. Here, we first assessed the clarity, strictness and timing of data- and code-sharing policies across 275 journals in ecology and evolution. Second, we assessed initial compliance to journal policies using submissions from two journals: *Proceedings of the Royal Society B* (Mar 2023–Feb 2024: *n* = 2340) and *Ecology Letters* (Jun 2021–Nov 2023: *n* = 571). Our results indicate the need for improvement: across 275 journals, 22.5% encouraged and 38.2% mandated data-sharing, while 26.6% encouraged and 26.9% mandated code-sharing. Journals that mandated data- or code-sharing typically required it for peer review (59.0% and 77.0%, respectively), which decreased when journals only encouraged sharing (40.3% and 24.7%, respectively). Our evaluation of policy compliance confirmed the important role of journals in increasing data- and code-sharing but also indicated the need for meaningful changes to enhance reproducibility. We provide seven recommendations to help improve data- and code-sharing, and policy compliance.

## 1. Introduction

In the last two decades, there has been a fundamental shift in the scientific community towards transparency; providing both data and code is now considered by many to be a minimum requirement for publication [1–3]. Indeed, some jurisdictions now require data to be shared, particularly as it originates from publicly funded research (e.g. the European Union’s Open Data Directive, 2019). As a result, journals have gradually moved towards implementing data- and code-sharing policies [4,5], with the number of journals that mandate data- and/or code-sharing greatly increasing in the last decade [1,6], including in ecology and evolution [5,7–10]. However, despite an increase in policy implementation in journals in recent years, compliance with these data- and code-sharing policies appears to be lagging [2,4,5,10–12]. As a result, the replicability and reproducibility of scientific findings remains low across numerous fields of research [11–16]. In part, such low compliance may be driven by vague language and non-definitive policy requirements [17], which can impede both author and editor understanding. Thus, we need to assess journal policies and their clarity of language to examine a potentially important cause of low compliance contributing to low reproducibility across ecology and evolution.

Whereas a large portion of research thus far has focused on the amount of data- and code-sharing post-publication (i.e. after acceptance), either in terms of journal policies or presence of data and code at the manuscript level, much less is known about the proportion of journals that require data and code upon manuscript submission (*during* peer review). Providing data and code for peer review not only allows for deeper insight into the manuscript for reviewers and editors [3,13,18], but can also promote reproducibility earlier in the publication process, as well as reduce the probability of errors in published papers [19–23] (but see [9,24]). Indeed, Heyard & Held [21] suggest that although preparing data and code for submission increases workload, it can promote the uptake of reproducible workflows in research groups, reduce sources of error, and improve the quality of the shared data and code. However, despite some journals adopting mandatory data- and code-sharing during peer review for some years (e.g. *Proceedings of the Royal Society B: Biological Sciences* in 2017, *The American Naturalist* in 2022 and *Ecology Letters* in 2023), little is known about the overall percentage of journals in ecology and evolution that have implemented any form of policy on data- and code-sharing for peer review and their associated compliance rates, when in place.

To this end, we first reviewed the current state of data- and code-sharing policies across 275 journals that publish studies in ecology and evolution. For each policy, we considered three main features: (i) the clarity of the policy (i.e. how easy it was to understand); (ii) the strictness of the policy (i.e. from mandatory to optional sharing); and (iii) the timing of the policy (i.e. at which point in the publication process data- and/or code-sharing was required or expected by the journal: during peer review or after acceptance). We aimed to test: (i) whether the number of journals encouraging or mandating data- and code-sharing ‘during peer review’ differed from the number encouraging or mandating sharing ‘after acceptance’; (ii) whether there are associations between data- and code-sharing strictness and timing, between a policy’s strictness and timing, and between a policy’s clarity and strictness; (iii) whether the number of journals mandating or encouraging data- and code-sharing (either ‘during peer review’ or ‘after acceptance’) has increased since the assessments by Berberi & Roche [9,25] (data) and Culina *et al*. [5] (code). More specifically, Berberi & Roche [9,25] found that out of 194 journals in ecology and evolutionary biology, only 35.6% (69/194) of journals mandated and 41.8% (81/194) encouraged data-sharing (using policies from the Living Database of Journal Policies in Ecology and Evolution [25], last updated in 2023). In contrast, out of 95 journals, Culina *et al.* [5] found that 75.8% (72/95) of journals mandated or encouraged code-sharing. Note that the total numbers described above for Berberi & Roche [9,25] and Culina *et al.* [5] represent the number of overlapping journals analysed in this paper. This study expands on the number of examined journals significantly and, importantly, investigates another aspect of journal policies: the timing of when data- and code-sharing is required.

Second, using data obtained directly from the editorial team at two journals that publish ecology and evolution studies (*Ecology Letters* and *Proceedings of the Royal Society B: Biological Sciences*), we examined: (i) whether the number of submissions sharing data and code differed from those that did not share; (ii) whether data-sharing was more common than code-sharing; and (iii) whether the introduction of a mandatory data- and code-sharing policy was associated with an increase in data- and code-sharing upon manuscript submission.

## 2. Methods

The pre-registration for this study is available at OSF [26] and was written after data collection but prior to the main data analysis on 21 May 2024. In addition, where appropriate, we provide author initials as per MeRIT to identify authors' roles in the methodology and elsewhere [27].

### (a) Statistical analysis

All analyses were conducted in R (v4.4.1; R Core Team, 2024 [28]). All data and code used for processing, analysis and visualization are available at Zenodo [29].

### (b) Data- and code-sharing policies

A list of journals that publish ecology and evolution studies was created by combining a series of previously generated lists (see electronic supplementary material, Methods) and resulted in a preliminary list of 284 journals after duplicates were removed. This was then used in the hackathon ‘Open code and data practices during peer review’, for policy extraction, which EIC, AS-T and NPM organized at the SORTEE conference on 17 October 2023. We removed nine journals from the list post-hackathon that were either duplicated or no longer appeared to be in operation (*n*_final_ = 275 journals; see electronic supplementary material, table S1).

Each journal was assigned to three separate data extractors (DEs, *n* = 36). Each DE was assigned an initial subset of 15 journals, with the option to extract additional subsets. Mean and median number of subsets of 15 journals assigned per person were 1.3 and 1, respectively (range = 1 to 3). For each journal, DEs extracted information regarding the timing of data- and code-sharing (categorical: not expected at all; during peer review; after acceptance (post-publication); unclear; other), policy strictness (categorical: not mentioned; encouraged; optional for authors; on reviewer request; mandated) and the clarity of the statement that describes the policy’s timing and strictness (via a semi-quantitative Likert scale with 5 levels: 1 = totally unclear, 5 = totally clear) by filling out a Google Form (full details on variables in electronic supplementary material, table S2). Between-DE agreement was tested using Fleiss kappa intraclass correlation scores for the categorical variables of strictness and timing, and Kendall’s W for the ordinal variable of clarity (via package {irr} v0.84.1; [30]).

Prior to analysis, we made a number of key methodological decisions regarding DE responses and up-to-date policies (see electronic supplementary material, ‘Preregistration Deviations’). This updated dataset was then used for the remaining analyses, including calculating several descriptive statistics (exploratory analyses listed in the pre-registration) and assessing the difference between journals in terms of code- and data-sharing strictness, timing and clarity. Specifically, NPM calculated Cramer’s V non-parametric correlations (i.e. between journal code- and data-sharing policy strictness; between journal code- and data-sharing timing; and, between strictness and clarity for both code and data separately; via package {confintr} v1.0.2 [31] as well as performed chi-squared tests (*χ*^2^) to assess if journals differ in whether code or data is expected ‘during peer review’ or ‘after acceptance’. Lastly, to assess changes in the number and percentage of journals mandating or encouraging data- or code-sharing, NPM compared our results with those of Berberi & Roche [9] and Culina *et al*. [5] using *χ*^2^ tests. To do this, we used the subset of overlapping journals between these studies and ours (Culina *et al*.: 95 out of 96 journals overlapped with our list [5]; Berberi & Roche: 194 out of 199 overlap [9]).

### (c) Journal-specific submission data

We received data related to the data and code submission for peer review on 28 February 2024 from *Proceedings of the Royal Society B: Biological Sciences* (hereafter *Proceedings B*), and on 2 April 2024 from *Ecology Letters*.

#### (i) Ecology Letters

For *Ecology Letters*, we received initial submission data for original research articles (i.e. ‘letters’) from two 3-month periods, Jun–Aug 2021 (i.e. the pre-mandate period, 280 submissions) and Sep–Nov 2023 (i.e. the post-mandate period, 291 submissions). Note that for *Ecology Letters,* the mandated enforcement of data- and code-sharing for peer review had been in place in August 2023. Seventy-nine out of the 280 submissions from the pre-mandate period were rejected before peer review, and no information on compliance could be extracted for these. Therefore, we only used data on the 201 remaining submissions during this period. During the pre-mandate period, authors were required to provide a data availability statement with their initial submission, either providing a link to the study’s data (e.g. via a digital object identifier (DOI), GitHub repository, or website) or stating that such a link would be provided upon acceptance (note that in the pre-mandate period, the requirement did not include any reference to code-sharing). The policy requiring a data availability statement had been in place since around 2018 (*Ecology Letters* editorial team, personal communication). Data from the pre-mandate period included whether a compliant data availability statement was provided, and if so, whether it included a link. During the post-mandate period, authors were required to submit a link (e.g. DOI, GitHub repository, or website) to the study’s data and code upon submission. This policy was implemented in early 2023 and has been systematically enforced by the managing editors since Aug 2023 (i.e. prior to any formal data editor or peer review, the managing editor was responsible for checking and enforcing the mandated policy). Data from the post-mandate period included whether a working link was provided upon first submission without verifying that all necessary data and code and associated metadata was provided. Note that letters submissions do not distinguish between research articles that may not rely on data or code (e.g. some theoretical papers); therefore, the percentage of non-compliant submissions may include a small number of submissions that did not use data or code.

With the data received from *Ecology Letters*, NPM assessed whether the frequency of policy-compliant submissions was higher than the frequency of non-policy-compliant submissions both within and between the pre- and post-mandate periods using *χ*^2^ tests. In addition, since compliance did not require the provision of a data-sharing link in the pre-mandate period, NPM also compared the frequency of submissions that did or did not provide a data-sharing link within the pre-mandate period, and between the pre- and post-mandate periods.

#### (ii) Proceedings of the Royal Society B: Biological Sciences

For *Proceedings B*, we received submission data for all article types from a single post-mandate period (i.e. Mar 2023–Feb 2024, 2340 submissions), where authors were required to provide data and/or code via sharing a link or uploading them as supplementary materials. This mandate has been in place since *ca*. 2017 (*Proceedings B* editorial team, personal communication). Data received included the manuscript type (e.g. research, evidence synthesis, comment, etc.) and the authors’ responses to the following submission questions: (i) 'Does your paper present new data, or use data/models published elsewhere?’; and (ii) 'If yes, provide a link to your data if it is in a repository. If depositing your data with Dryad, ensure that you give the private reviewer a "sharing" link. If your data is uploaded as supplementary material, please state this. Your paper will be unsubmitted without this information.’ Note that although the *Proceedings B* policy requires data- and code-sharing, the two questions above only referred to data.

By contrast to *Ecology Letters*, we did not have access to pre-mandate data, and therefore could not assess policy compliance or the percentage of submissions providing a data- and/or code-sharing link for *Proceedings B* before the mandate. In addition, we were not able to assess initial policy compliance because submissions determined to be non-compliant by the managing editors were unsubmitted and authors were required to add the missing information before resubmitting. Therefore, we first used question (i) above to explore the proportion of papers from each manuscript type that had data associated with them. Then, using the authors’ response to question (ii) above for the subset of research manuscripts with associated data (i.e. 2000 submissions), NPM compared the percentage of submissions that appeared to have provided a data- and/or code-sharing link to those that provided data and/or code as supplementary materials. In addition, NPM estimated the percentage of submissions that appeared to have provided data, code, or both data and code, by categorizing them based on the text provided by the authors in question (ii), and compared the proportion appearing to share data to the proportion appearing to share code using *χ*^2^ tests.

## 3. Results

### (a) Code- and data-sharing policies

Overall, fewer than a quarter of all 275 investigated journals implemented ‘mandated data-sharing during peer review’ (22.6%; table 1; electronic supplementary material, figure S1), however, this was still the most common data-sharing policy. The second and third most common policies were data-sharing on ‘reviewer request during peer review’ (17.1%) and ‘mandated data-sharing post-publication’ (15.6%). Lastly, 10.6% of all journals did not have any form of data-sharing policy (table 1; electronic supplementary material, figure S1). Regarding code, the most common finding was for journals to have no code-sharing policy (23.3%; table 1; electronic supplementary material, figure S1). For journals that did have a code-sharing policy, the percentages were similar to data-sharing, where the most common policy was mandated code-sharing during peer review (20.7%) closely followed by encouraged code-sharing post-publication (20.0%) and code-sharing on reviewer request during peer review (18.6%; table 1; electronic supplementary material, figure S1). In total, 167 journals mandated or encouraged data-sharing (60.7%). A similar proportion of these, about half, required some kind of data-sharing during peer review (87, 52.1%) or during post-publication (80, 47.9%; *χ*^2^ = 0.293; *p* = 0.588). The results were similar for code-sharing: of the 147 journals that mandated or encouraged code-sharing (53.5%), a similar percentage required it during peer review (75, 51.0%) than during post-publication (72, 49.0%; *χ*^2^ = 0.061; *p* = 0.805).

**Table 1.** Summary of policy requirements for data- and code-sharing for 275 ecological and evolutionary journals. Note that 'not expected at all' under 'not mentioned' indicates no policy, and 'not expected at all' under 'optional for authors' indicates there is no requirement for new sharing of ‘new’ data or code, or a journal simply wants an availability statement (see ‘Methodological clarifications’ above).

policy strictness	policy timing	data policy	code policy
mandated	— during peer review	62 (22.55%)	57 (20.73%)
	— after acceptance (post-publication)	43 (15.64%)	17 (6.18%)
encouraged	— during peer review	25 (9.09%)	18 (6.55%)
	— after acceptance (post-publication)	37 (13.45%)	55 (20.00%)
on reviewer request	— during peer review	47 (17.09%)	51 (18.55%)
optional for authors	— during peer review	15 (5.45%)	2 (0.73%)
	after acceptance (post-publication)	9 (3.27%)	11 (4.00%)
	not expected at all	8 (2.91%)	0 (0.00%)
not mentioned	not expected at all	29 (10.55%)	64 (23.27%)

We also found a statistically significant correlation between the strictness of data-sharing policies and the strictness of code-sharing policies (*V* = 0.546, 95% confidence interval (hereafter 95 CI): [0.489, 0.605]), consistent with journals with stricter data-sharing policies tending to have stricter code-sharing policies. Similarly, the timing of both data- and code-sharing requirements were also statistically significantly correlated (*V* = 0.733, 95 CI: [0.651, 0.817]). Also, the strictness and clarity rating of a policy were statistically significantly correlated both for data (*V* = 0.295, 95 CI: [0.230, 0.366]) and code (*V* = 0.217, 95 CI: [0.163, 0.281]). Summary statistics show that that highest mean clarity rating was for journals with mandated data-sharing (3.77, SD = 0.99; median = 4) compared with the overall mean (3.49, SD = 1.16; median = 4) and for journals with mandated code-sharing (3.08, SD = 1.25; median = 3) compared with the overall mean (2.56, SD = 1.33; median = 2), although the difference between mandated journals and the overall means were relatively small.

The number of journals that mandate data- and code-sharing has increased. In 2023, Berberi & Roche [9,25] found that 35.6% of journals (*n* = 69 out of 194) mandated data-sharing, compared with 41.2% in 2024 (*n* = 80). They also found that 41.8% of journals (*n* = 81) had a weak data-sharing policy and 22.7% (*n* = 44) appeared to have no data-sharing policy at all. In comparison, we found that 49% of journals (*n* = 95) had a non-mandated or weak data-sharing policy (i.e. encouraged, on reviewer request, optional), and only 9.8% of journals (*n* = 19) appeared to have no data-sharing policy at all. While the results do not represent a statistically significant increase in the percentage of journals in this subset that mandate data-sharing between 2023 and 2024 (*χ*^2^ = 1.090; *p* = 0.297), there was a statistically significant increase in the percentage of journals with some form of data-sharing policy during this period (*χ*^2^ = 10.915; *p* < 0.001). Culina *et al*. [5] found that 72 out of 95 journals (75.8%) encouraged or mandated code-sharing in 2020. Here, we found that 84 out of those 95 journals (88.4%) now have implemented some code-sharing policy. This includes journals that we classified as ‘on reviewer request’ (*n* = 21). This difference corresponds to a statistically significant increase in the percentage of journals implementing some form of code-sharing policy between 2020 and 2024 (*χ*^2^ = 4.334; *p* = 0.037). Incorporating three additional journals that we had categorised as ‘optional for authors’ increases this percentage to 91.6%, which would represent an even greater increase since 2020 (*χ*^2^ = 7.555; *p* = 0.006). Note that the observed increase may be partially influenced by subtle differences in the categorizations used between Culina *et al*. [5] and here.

The agreement between DEs was statistically significant, suggesting there was general agreement on the timing, strictness, and clarity of the data- and code-sharing policies for journals (table 2). The percentage of full disagreement (i.e. all DEs gave different responses) ranged from 7% for data strictness to 30% for both data and code clarity (table 2). Although disagreement appeared to be relatively higher for clarity scores, this is not surprising given clarity is based on a Likert scale of the DEs perceptions of the journals policy statements, rather than the actual policy, so it likely incorporates a greater level of subjectivity. Nonetheless, the agreement coefficients support moderate to strong levels of interrater agreement for clarity.

**Table 2.** Summary agreement data between data extractors (DEs) for policy timing, strictness and clarity for data- and code-sharing submission policies across journals that publish ecology and evolution studies. Agreement scores show full agreement (3 out of 3 DEs scored the journal the same), partial agreement (2 out of 3 DEs scored the journal the same) and no agreement (i.e. all DEs scored the journal differently). The analysis of timing (‘during peer review’, ‘after acceptance’, ‘not expected at all’, ‘unclear’) and strictness (‘encouraged’, ‘optional’, ‘not mentioned’, ‘on reviewer request’, or ‘mandated’) includes all 275 journals, whereas that of clarity (from 1−5) includes 246 journals for data and 211 journals for code, which corresponds to the subset of journals mentioning data- and/or code-sharing in their policies.

response		full agreement (3/3)	partial agreement (2/3)	no agreement (0/3)	between-DE agreement coefficients
timing	— data	71 (25.82%)	167 (60.73%)	37 (13.45%)	Fleiss's *κ*: 0.261 (*p* < 0.001)
	— code	90 (32.73%)	142 (51.64%)	43 (15.64%)	Fleiss's *κ*: 0.259 (*p* < 0.001)
strictness	— data	156 (56.73%)	100 (36.36%)	19 (6.91%)	Fleiss's *κ*: 0.555 (*p* < 0.001)
	— code	118 (42.91%)	123 (44.73%)	34 (12.36%)	Fleiss's *κ*: 0.389 (*p* < 0.001)
clarity	— data	29 (11.79%)	142 (57.72%)	75 (30.49%)	Kendall’s *W*: 0.505 (*p* < 0.001)
	— code	34 (16.11%)	114 (54.03%)	63 (29.86%)	Kendall’s *W*: 0.535 (*p* < 0.001)

### (b) Journal-specific submission data

#### (i) Ecology Letters

The *Ecology Letters* manuscript submission data showed that in the pre-mandate period (i.e. where the authors were required to simply provide a data availability statement in their initial submission) the number of submissions complying with the policy was statistically significantly higher than the number of non-compliant submissions (tables 3 and 4). By contrast, in the post-mandate period (i.e. where the authors were required to provide a link to the study’s data and/or code in their initial submission) policy compliance was statistically significantly lower than non-compliance (tables 3 and 4). Nonetheless, despite lower policy compliance during the post-mandate period, the percentage of submissions including a link to the study’s data and/or code increased significantly after the introduction of the mandate (tables 3 and 4; however, we note that we could neither assess compliance for data- and code-sharing separately nor confirm whether all data and code were provided). Fewer submissions appeared to include data and/or code with their submission (i.e. provided a link) than those that did not, in both the pre-mandate and post-mandate period (tables 3 and 4).

**Table 3.** *Ecology Letters* submission data summary. There are 201 submissions with data from the pre-mandate period and 291 submissions with data from the post-mandate period. Pre-mandate compliance requires submissions to include a data availability statement, which may or may not include a link to the study’s data, while post-mandate compliance requires submissions to include an active link to a data/code repository of the study.

was the submission apparently compliant with the period-specific policy at the time?	pre-mandate (2021) (*policy: data-sharing statement*)	post-mandate (2023) (*policy: data/code-sharing link*)
— yes	134 (66.67%)	124 (42.61%)
— no	67 (33.33%)	167 (57.39%)

did the submission include a link to the study’s data and/or code?		
— yes	34 (16.92%)	124 (42.61%)
— no	167 (83.08%)	167 (57.39%)

**Table 4.** Comparisons of compliance and link sharing within and between pre- and post-mandate periods for *Ecology Letters* submissions.

pre-mandate period (2021)	*χ*^2^ (*p*‐value)
— compliance is higher than non-compliance.	22.333 (*p* < 0.001)
— fewer submissions included DOI/ links than those that did not.	88.005 (*p* < 0.001)

post-mandate period (2023)	
— non-compliance is higher than compliance, and fewer submissions included DOI/ links than those that did not.	6.354 (*p* = 0.012)

pre- versus post-mandate period (2021 versus 2023)	
— compliance was lower in the post-mandate period.	26.626 (*p* < 0.001)
— link sharing was higher in the post-mandate period.	34.838 (*p* < 0.001)

#### (ii) Proceedings of the Royal Society B: Biological Sciences

*Proceedings B* submission data showed that the manuscript types ‘Research’ and ‘Evidence Synthesis’ had the highest percentage of submissions with associated data (90.3% and 81.3%, respectively; table 5), however note that this does not infer whether the associated data and/or code was *actually* shared with the manuscript. Based on the authors’ responses to the two submission questions, the percentage of submissions that appear to have included a link to and/or uploaded the data and/or code as supplementary material was very high across all manuscript types (96.5%; table 6). Many of the submissions that did not provide data or code via a link or supplementary material were genetic studies that included only accession numbers to public sequence repositories (e.g. GenBank), which are currently treated as policy-compliant during the submission screening process. More articles chose to share their data and/or code via a link rather than as supplementary material (table 6). Lastly, a considerable percentage of submissions appear to share only data (45.1%), followed by submissions sharing both data and code (29.5%), or only code (3.3%). As a result, the number of papers appearing to share data compared with code was statistically significantly higher (*χ*^2^ = 675.79; *p* < 0.001; table 6). Submissions mentioning code only often corresponded to simulation- or computation-based studies.

**Table 5.** Summary data for the submission question (i): ‘Does your paper present new data, or use data/models published elsewhere?’ by manuscript type for submissions to *Proceedings B*. Note that the column ‘My paper contains data’ has been adjusted from the original question (it was simply ‘yes’). Values in parenthesis relate to percentage.

manuscript type	‘my paper has no data’	*‘*my paper contains data’
research	216 (9.75%)	2000 (90.25%)
review	47 (73.44%)	17 (26.56%)
biological science practices	7 (36.84%)	12 (63.16%)
evidence synthesis	3 (18.75%)	13 (81.25%)
commentary	10 (90.91%)	1 (9.09%)
special feature reviews	7 (87.50%)	1 (12.50%)
invited reply	2 (66.67%)	1 (33.33%)
comment	2 (100.00%)	0 (0.00%)

**Table 6.** Summary data for how data and/or code is provided for original research type manuscripts (total submissions = 2000), and the apparent levels of data, code or data- and code-sharing based on the statement provided by authors in response to the submission question (ii) for submissions to *Proceedings B*: ‘If yes, provide a link to your data if it is in a repository. If depositing your data with Dryad, ensure that you give the private reviewer a ‘sharing' link. If your data is uploaded as supplementary material, please state this. Your paper will be unsubmitted without this information.'

is a link and/or supplementary materials apparently provided?	
— yes	1929 (96.45%)
— no	64 (3.20%)
— unclear	7 (0.35%)

if it is provided, how?	
— link	1094 (56.71%)
— supplementary materials	713 (36.96%)
— both	121 (6.27%)

based on the author’s response, does data, code or both appear to be provided?	
— data only	869 (45.05%)
— code only	63 (3.27%)
— data and code	568 (29.45%)
— unclear	428 (22.19%)

## 4. Discussion

We had two overall aims for this study: (i) to assess the current state of data- and code-sharing policies in journals that publish ecology and evolution studies; (ii) to assess the influence of journal policy mandates on data- and code-sharing compliance using initial manuscript submission data obtained directly from the editorial offices of two journals. Our results show that uptake of data- and code-sharing policies in ecology and evolution is slow; less than half of all journals possess some form of mandated data- or code-sharing policy. Of these, even fewer journals facilitate data and code review by requiring authors to share data and code during peer review. Once such a mandate is in place, it appears to be followed by higher rates of data- and code-sharing, despite low initial compliance. We argue that some reasons for low compliance stem from a lack of journal enforcement of mandated policies (see [32]) in addition to the wide variety of data- and code-sharing policies that are often unclear and difficult-to-interpret or may be inconsistent between journals and associated publishers.

Across all 275 journals that publish ecology and evolution studies, we found that, in 2024, only 38.2% mandated data-sharing either during peer review or after acceptance, and 10.6% did not even mention data in their policies. The remaining 51.2% either encouraged data-sharing (22.5% of all journals), only required it upon explicit reviewer request (17.1%), or made it optional for authors (11.6%). Mandating code-sharing was substantially lower compared with data-sharing (26.9%) with about a quarter of the journals (23.3%) not mentioning code in their policies. The remaining half (49.5%) either encouraged code-sharing (26.6%), only required it upon reviewer request (18.6%), or made it optional for authors (4.7%). These results are in agreement with previous findings showing that code-sharing policies are far less common than data-sharing policies [1,5–7]. It should be noted that the number of journals encouraging or mandating data and code during peer review in our study may likely be an overestimation due to the large proportion of policies originating from a single publisher, where we had decided to be particularly lenient in our policy timing categorisation (ScienceDirect by Elsevier; mandated: 31 journals, encouraged: 7 journals). In this specific case, we had taken the policy to refer to data- and code-sharing ‘during peer review’, despite no specific reference in text (see details in electronic supplementary material, ‘Methodological Clarifications’). As such, our percentages likely reflect ceiling values (i.e. a best-case scenario). If we replace these ‘during peer review’ policy timings with ‘after acceptance’, the percentage of journals mandating data- and code-sharing during peer review would be halved (i.e. 11.2% and 9.5%, respectively) and, if we considered encouraging data- and code-sharing during peer review, they would be reduced by a third (i.e. 6.5% and 4.0%, respectively). These are all worrying statistics.

When a policy was in place, we found evidence that data- and code-sharing policies are typically aligned with one another in terms of both timing and strictness. Journals with stricter data-sharing policies tend to have stricter code-sharing policies and require data- and code-sharing at similar stages. These results are in line with previous surveys in ecology and evolution that have found that articles that shared data were up to 12 times more likely to share code than articles that did not share data [33]. Importantly, we also found evidence that the average clarity of policy was related to strictness, with journals that mandated data- and code-sharing having policies that were clearer to understand and to locate when compared with the average policy clarity among journals. These results are particularly important when considering previous findings, suggesting that policy wording is an important factor in aiding authors (and even editors) interpretation [17]. These findings underscore the imperative for journals to make their data- and code-sharing policy wording as clear and easy-to-find as possible, so as to increase author and editor understanding and, thus, aid policy compliance.

Our analysis reinforces positive trends found in previous meta-research studies, which demonstrate slow but steady improvement in the state of data- and code-sharing in ecology and evolution. The number of journals mandating data-sharing for the subset of 194 journals surveyed by Berberi & Roche [9,25] has increased from 36% to 41%, since 2023. Importantly, while still non-zero, the number of journals with no data-sharing policy in the same subset has decreased substantially from 23% to 10% (i.e. a 57% decrease since 2023). For code, the number of journals mandating or encouraging code-sharing for the smaller subset of 95 journals investigated by Culina *et al*. [5] also increased from 76% in 2020 to 89% in 2024. However, this increase is seemingly slowing. Mislan *et al*. [7] found that only 15% of those 95 journals had a code-sharing policy in 2015, thus, from an average increase in 12 journals/year adding a code-sharing policy from 2015 to 2020, the increase has slowed down to 3 journals/year from 2020 to 2024. Although data-, and particularly code-, sharing remains generally low in ecology and evolution, with recent meta-research studies suggesting rates between 12% and 79% for data-sharing, and between 3% and 27% for code-sharing (e.g. 2001−2013 [34]; 2015−2019 [5]; 2010−2019 [35]; 2015−2017 [10,11]; 2010−2022 [33]; 2015−2019 [36]), both are nonetheless increasing [5,10,33,36]. This study builds on previous findings and illustrates that more still needs to be done to highlight the importance of data- and code-sharing, particularly in relation to enhancing long-term reproducibility by increasing data and code availability [11,34].

Our results also indicate that journals have significant power to contribute to increased data- and code-sharing. In *Ecology Letters*, the number of submissions providing a link to data and/or code increased in the post-mandate period, despite the number of submissions adhering to the policy decreasing between the pre- and post-mandate period. Though perhaps counterintuitive at first, this pattern makes logical sense and is unsurprising if the barrier to compliance upon initial submission is higher (i.e. data and code were required upon first submission in 2023 compared with simply requiring a data availability statement in 2021) and authors fail to provide the required files upon initial submission. The increase in the number of submissions providing links to data and/or code in the post-mandate period is suggestive of an overall increase and a positive effect of the policy implementation (in addition to editorial policing) by *Ecology Letters*. It is important to note that using *initial* policy compliance upon submission avoids the potentially confounding influence of data editors, which has been in place in *Ecology Letters* since 2023 [37] and which actively contributes to increased policy compliance and reusable data and code, typically at a later stage in the peer review process. However, these data do not allow us to account for the overall increase in rates of data- and code-sharing over time (reflecting a broader cultural shift towards more open and reproducible science), which may have contributed to the increased number of submissions providing data and/or code.

Nonetheless, it does appear that when these policies are enforced and have been established for several years, the number of submissions providing data is very high. For *Proceedings B*, we found a level of compliance close to 100% within the submission period (96.5% in Mar 2023–Feb 2024), but note that we did not have access to *initial* submissions that may have been previously unsubmitted by the editorial office for not adhering to the data- and code-sharing policy. Thus, we are likely overestimating the effect of the policy mandate owing to editorial enforcement (although conversely it shows the substantial positive effect of successfully enforcing a mandate). Nevertheless, most submissions stated that they had provided data (74.5%), which was far greater than those providing code (32.7%). This discrepancy may be partially explained by the submission system focusing on data-sharing without explicitly asking authors to share their code. Therefore, although data-sharing compliance appears high in *Proceedings B*, code-sharing still lags behind, as observed in previous surveys [12]. We note, however, that we were unable to answer two of the pre-registered hypotheses related to the journal-specific submission data due to the type of submission data obtained from both journals. Specifically, for *Ecology Letters*, we were not able to separate whether rates of data- and code-sharing differed. For *Proceedings B*, we only obtained data from a post-mandate period, so we were unable to test the effects of the introduction of a data- and code-sharing policy on rates of submission compliance, and the data we obtained did not include manuscripts unsubmitted for not initially adhering to the policies. Nonetheless, our analyses clearly show the great potential of manuscript submission data provided by journals for understanding research practices, the efficacy of journal policies, and how to improve them, underscoring collaboration with journals as essential. Ensuring the recording and transparency of a journal’s submission data, and treating this as scientific data in its own right, should be of high priority as it provides an invaluable resource for the science of science (i.e. meta-research).

One thing is clear though, if journals implement data- and code-sharing policies, the overall availability of data and code increases, even when policy compliance is far from ideal [11,34,38–43]. Our results highlight that there is much room for improvement by journals to ensure the long-term reproducibility of scientific findings. In particular, the high percentage of journals failing to mention data- or code-sharing in their policies is concerning given that both data and code are key research products that not only increase the reproducibility and reliability of results but also their credibility (e.g. [44,45]) and impact (measured here as a citation advantage for data-sharing: [33,46–48]; and code-sharing: [49–51]; but see [52]. However, it should be noted that the presence of data and code and associated policies on sharing does not guarantee that archived material will be of sufficient quality to assess reproducibility [10,53]. Therefore, not only is it important to implement mandated sharing, it is also important that the archived data, code and associated metadata are of sufficient quality to meet the minimum standards of reproducibility; fortunately, not only do multiple detailed guides exist that can help with this [6,23,54–57] but many journals (including the two in this manuscript) employ dedicated data editors to ensure that data and code meet certain standards and adhere to proposed principles and guidelines (FAIR: findable, accessible, interoperable and reusable [58,59]; TADA: transferable, accessible, documented and annotated [55]; see also [60]).

### (a) Advice for journals

**Figure 1.**
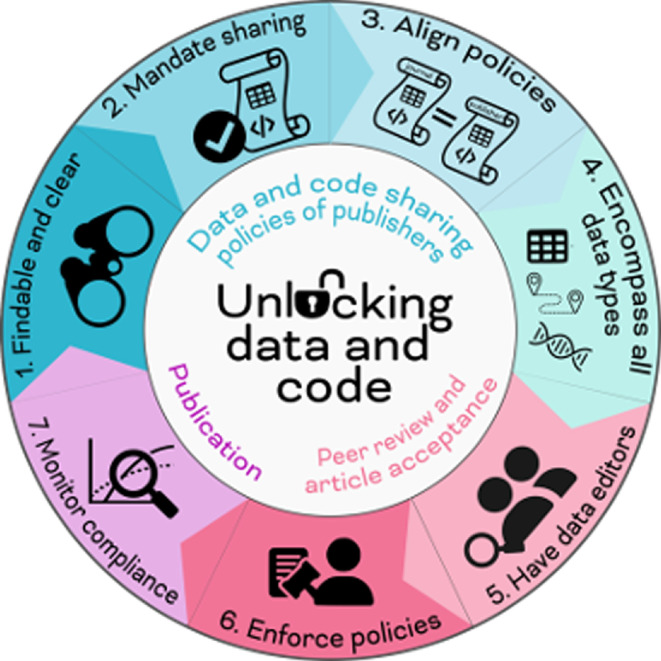
Visual depiction of the 7 points of advice for journals integrated in the publication process. Suggestions 1−4 involve the journal and/or publisher, suggestions 5–6 occur during peer review and article acceptance, and suggestion 7 occurs after publication to monitor the success of the process. Icons modified from Flaticon: table-grid by Dave Gandy, coding by Major Icons, regulation by IwitoStudio, people by Muhazdinata, copy by torskaya, magnifier by Creative Stall Premium. Figure by R.Z. and K.R.B.-N.

Our assessment of sharing policies across journals that publish ecology and evolution studies has given us insight into potential areas of improvement for data- and code-sharing policies. We list seven points of advice in figure 1.

(1) *Develop explicit, unambiguous and easy-to-find policies*. Ambiguity in journal requirements can lead to confusion and poor compliance [17]. Terms used in the policy should be clearly defined with clear guidance on the timing and specific requirements for data- and code-sharing. For instance, what constitutes ‘*complying with field standards’* (‘All authors are requested to make sure that all data and materials as well as software application or custom code support their published claims and comply with field standards’), *‘novel code’* or ‘*new simulations or analytical methods*’ (‘Novel code must be supplied as private-for-peer review in an external repository during the review process’; ‘Where a paper describes new simulations or analytical methods, we require authors to make any relevant software publicly available, wherever possible’), *‘some types of data’* (‘We require some types of data to be provided in manuscripts or deposited in public, community-supported repositories, prior to publication’) or *‘when- or wherever possible’* (‘We suggest that data be presented in the main manuscript or additional supporting files, or deposited in a public repository whenever possible’). Note, the text has been adjusted to maintain the anonymity of journals.
  For instance, instructions could include (under a section clearly labelled within author instructions): ‘All data and coding scripts used in the analysis to produce the results of the submitted manuscript must be archived prior to submission in a publicly available repository which produces a DOI (or another globally unique and persistent identifier) and directly adheres to principles and guidelines (FAIR, TADA).’
  The text above represents the bare minimum of requirement for a journal. We cannot provide an exhaustive list of requirements, rather we urge journals to be explicit about what is required and when, and detail this in an easy-to-find place for authors.
  It will then be up to the journal to decide what form of data is required. Although ideally raw data would be provided, this may not always be possible, particularly if the raw data is either in a non-digital or in an extremely large form or represents sensitive non-shareable data. We suggest journals move away from allowing data and code (and associated metadata) to be provided within the supplementary material and mandate storage within appropriate repositories. If uploaded within a PDF or word file, data and code cannot be readily opened and used without needing to convert to another file format prior to use. In addition, if copied and pasted, there is a risk of including unwanted formatting or characters in the resulting code or data. Most importantly, storage within supplementary material does not ensure long-term persistence and openness. A journal could decide at any time to remove the supplementary material (or webpage associated with that article) and all associated data and code would be lost. Furthermore, access to the supplementary material relies on the article being fully open access or the reader having a subscription to the specific journal, storage within a public repository removes this potential barrier to data- and code-sharing. Lastly, if analysis is done using a graphical user interface (such as SPSS or Minitab), scripts should still be exported and uploaded from these programmes, or at the very least, screenshots or a detailed text walkthrough provided for users to replicate the analyses.
(2) *Mandate sharing of both data and code during peer review*. There are numerous benefits to providing both data and code during peer review including early error detection, increased understanding of experimental and statistical methods, and the ability to assess the computational reproducibility and general reliability of the results during peer review. Sharing during peer review may also encourage authors to prepare their data and code in a way that is both understandable and reusable. Furthermore, data and code are often promised ‘upon request’, but this promise is rarely fulfilled [61,62]. Journals should move away from ‘request-based’ policies and instead require direct and mandated deposition of materials. This shift would eliminate ambiguity and make it easier to track and enforce compliance.
(3) *Align journal- and publisher-level policies*. In several cases, there was variability between when the journal expected data- and code-sharing and when the publisher expected data- and code-sharing (for instance, between *Springer Nature* journal and publisher policies). These should remain consistent in terminology (in strictness and timing) and, if possible, be specifically referenced in the journal’s author guidelines to reduce confusion.
(4)*Ensure that policy applies to all types of data and code*. Journals should mandate data- and code-sharing for all types of data and code rather than only certain types (e.g. all data should be mandated rather than simply DNA or protein sequences. Similarly, requiring sharing of all computer code, rather than simply ‘custom code’ which may lead to confusion due to ambiguity). This would make the policy less ambiguous, and improve the general reproducibility of all the results, rather than just some. Although we note that there may be exceptions to this rule in rare circumstances (e.g. sensitive personal information or data involving endangered species; see [54]). Journals should ensure that this is clearly mentioned and specified under their data- and/or code-sharing policy section.
(5) *Have data editors.* To assess the adherence of data and code to FAIR principles and other guidelines, some journals use the expertise and knowledge of dedicated data editors or reviewers (e.g. STAR checks in *Psychological Science*; see [63]). While this might require additional resources and would not affect policy compliance upon *initial* submission, it can significantly enhance the rigour of the review process and the reproducibility of the final published study. As a result we encourage all journals to consider data editors as part of their formal review process.
(6) *Enforce the policy.* Journals should enforce their mandated policies by first checking author compliance [64]. This can be done at a basic level by checking if data or code links are provided; higher-level checks may require dedicated staff or software (e.g. DataSeer.ai, https://dataseer.ai/) at journals (see below). Journals should clearly state the consequences of not adhering to initial data- and code-sharing requirements. These consequences could range from simply having to resubmit the manuscript with accompanying data and/or code during initial submission, to rejection of the manuscript, or to publication of a note highlighting the lack of data and/or code availability. A clear articulation of consequences would motivate authors to comply. Although the most suitable piece of advice is for journals to hire dedicated data editors to assess data and code quality (see above), reviewers could either note in their review whether data and code was available for peer review or alternatively, return the manuscript if authors have not complied with mandated sharing. While we do not expect reviewers to be able to run detailed checks akin to a data editor, at the very least they could check whether data and code is provided and adhering to the journal policy.
(7) *Continuous monitoring and evaluation of policy compliance rates.* Journals should carefully curate their submission data and regularly monitor and openly report on compliance rates to their data- and code-sharing policies. This data can be used to identify areas where policies need to be improved or enforcement needs to be strengthened. It also allows funding agencies and institutions to track good scientific practice and institutional data policies, which nowadays more often include mandatory and open data- and code-sharing (such an initiative is already being conducted at *PLoS*, *Open Science Indicators* [65]).

## Data Availability

All data and code used for processing, analysis and visualization are available at Zenodo [29]. Supplementary material is available online [66].
